# Anisotropic sensor and memory device with a ferromagnetic tunnel barrier as the only magnetic element

**DOI:** 10.1038/s41598-017-19129-5

**Published:** 2018-01-16

**Authors:** L. Lόpez-Mir, C. Frontera, H. Aramberri, K. Bouzehouane, J. Cisneros-Fernández, B. Bozzo, L. Balcells, B. Martínez

**Affiliations:** 1 Institut de Ciència de Materials de Barcelona (ICMAB-CSIC), 08193 Bellaterra, Spain; 2 Unitè Mixte de Physique, CNRS-Thales, Palaiseau, 91767 France

## Abstract

Multiple spin functionalities are probed on Pt/La_2_Co_0.8_Mn_1.2_O_6_/Nb:SrTiO_3_, a device composed by a ferromagnetic insulating barrier sandwiched between non-magnetic electrodes. Uniquely, La_2_Co_0.8_Mn_1.2_O_6_ thin films present strong perpendicular magnetic anisotropy of magnetocrystalline origin, property of major interest for spintronics. The junction has an estimated spin-filtering efficiency of 99.7% and tunneling anisotropic magnetoresistance (TAMR) values up to 30% at low temperatures. This remarkable angular dependence of the magnetoresistance is associated with the magnetic anisotropy whose origin lies in the large spin-orbit interaction of Co^2+^ which is additionally tuned by the strain of the crystal lattice. Furthermore, we found that the junction can operate as an electrically readable magnetic memory device. The findings of this work demonstrate that a single ferromagnetic insulating barrier with strong magnetocrystalline anisotropy is sufficient for realizing sensor and memory functionalities in a tunneling device based on TAMR.

## Introduction

The ever-increasing demand for storage and high speed processing of large amounts of data boost the search for new materials/paradigms for improving current microelectronics and magnetic storage technologies and put forward spintronics as a promising technology. Thus, spintronics has become one of the most active areas of research in condensed mater physics during the last decade because of its attractive prospects of faster and lower energy consumption electronic devices. Since spintronics is based on the use of the spin of the electrons as a control variable, the generation and control of highly spin-polarized currents is a fundamental issue. The most straightforward way to get spin polarized currents is to use ferromagnetic metals. However, the spin polarization degree (≈40%) achievable with these materials is simply too low to obtain high-performance competitive devices^1^. Fully spin polarized currents can be obtained using some complex oxides exhibiting half metallic character, i.e. full spin polarization, although their relatively low resistances compromise their use as spin injectors^2,3^. Another versatile way is through spin filtering. This effect consists in spin selective tunneling through a barrier sensitive to spin orientation. In contrast to a non-magnetic insulator, a ferromagnetic insulating (FM-I) barrier provides two different barrier heights for spin-up and spin-down electrons^4^. Since the tunneling probability depends exponentially on the barrier height, spin selection can be very efficient in FM-I-based junctions. In this context, FM-I materials have attracted great interest over the past years as spin sources and spin filters^2,5^. However, FM-I materials are scarce in nature as ferromagnetic interactions are typically of exchange-type mediated by charge carriers. Eu chalcogenides were the first materials used as FM-I barriers convincingly demonstrating spin filtering tunneling effects in magnetic tunnel junction (MTJ)-like structures, even though they suffer from very low ferromagnetic transition temperatures^4,6^. On the search for magnetic (either ferromagnetic or ferrimagnetic) insulating materials few perovskites (BiMnO_3_)^7^ and various spinels (NiFe_2_O_4_^8^, MnFe_2_O_4_^9^, NiCo_2_O_4_^10^, CoFe_2_O_4_^11^, and CuCr_2_O_4_^12^) have been tested for spin-filtering purposes. In most of these cases, spin polarization is measured through Julière’s method by collecting the transmitted current with a ferromagnetic electrode on top of the FM-I barrier. The Meservey-Tedrow technique is another way used to measure the spin polarization employing a superconductor instead of a ferromagnetic collector^11^. Some other works exploit the magnetic field dependence of the tunneling current through the barrier formed in ferromagnet/semiconductor-electrode systems, e.g. Fe_3_O_4_/Nb:SrTiO_3_ and *γ*-Fe_3_O_4_/Nb:SrTiO_3_ systems^13–15^. In these cases the two-current model is used to estimate the spin filtering efficiency. This model assumes two differentiated tunneling channels for the spin-down and spin-up electrons and an additional splitting of the bands due to a Zeeman term when an external magnetic field is considered.

In the present work, we build a tunnel junction based on La_2_Co_0.8_Mn_1.2_O_6_ (LCMO) grown on semiconducting electrode Nb:SrTiO_3_ (Nb:STO). Nb:STO is a widely used conducting substrate which enable good epitaxy with a large number of oxides, such as La_0.9_Ca_0.1_MnO_3+δ_/Nb:STO^16^ or La_0.7_Sr_0.3_MnO_3_/Nb:STO^17,18^ manganite systems. LCMO is a double perovskite exhibiting FM-I behavior and a relatively high Curie temperature (*T*_*C*_) of 230 K. Perovskites have a less complex structure than spinels, and seem to be more appropriate for device implementation. In previous works we have shown that high quality LCMO epitaxial thin films can be prepared by sputtering with a *T*_*C*_ ≈ 225K and a saturation magnetization (*M*_*S*_) of about 6 *μ*_*B*_/f.*u*., which suggest a high degree of Co/Mn cationic ordering^19,20^. We have also shown that LCMO grown under tensile strain (on STO) exhibits a strong perpendicular magnetic anisotropy (PMA), which has a magnetocrystalline origin due to the spin-orbit coupling (SOC) in Co^2+^ ions^20,21^. This suggests that LCMO is a good candidate to exhibit tunneling anisotropic magnetoresistance (TAMR). TAMR measures the dependence of spin-polarized electron transport on the orientation of the magnetization with respect to the crystallographic axes^22,23^ and arises from SOC. In fact, theoretical works have predicted that TAMR is a generic effect in ferromagnetic metals with SOC, induced by the dependence of density of states of tunneling electrons on the orientation of the applied magnetic field^24^. TAMR, unlike conventional tunneling magnetoresistance, can be found in tunnel structures with only one magnetic electrode (metallic or semiconductor). Thus far, it has been proved in (Ga,Mn)As/AlO_*x*_/Au^25^, Fe/GaAs/Au^26^ and CoPt/AlO_*x*_/Pt^27^ tunnel structures. In this work we show that TAMR effects can arise in a device with a FM-I barrier as the only magnetic component. Exploiting TAMR in NM/FM-I/OS (NM = non-magnetic metal, OS = oxide semiconductor) structures that could operate without magnetic electrodes represents an alternative of much easier technological implementation than conventional MTJs, avoiding the need of two magnetically decoupled ferromagnetic electrodes and coherent tunneling.

## Results and Discussion

### Analysis of the transport through the barrier

**Figure 1:**
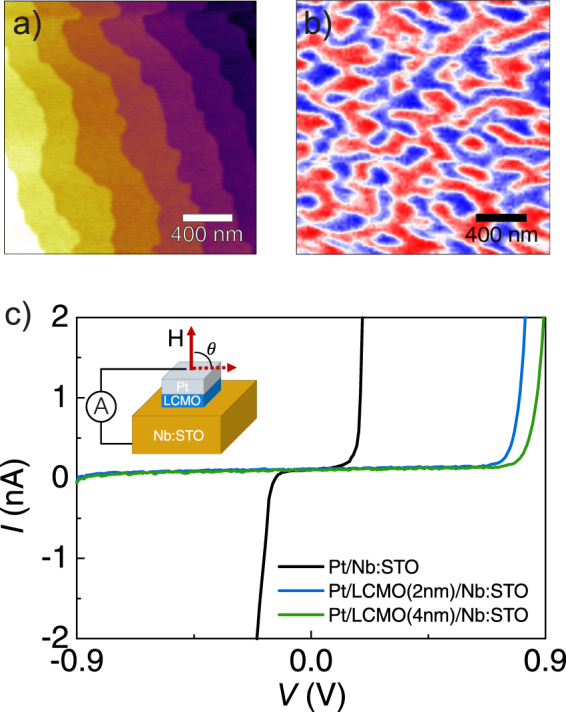
(**a**) Topography and (**b**) MFM image of Pt/4nm-LCMO/Nb:STO. MFM is measured at 77 K after zero-field cooling. An equal distribution of up (blue) and down (red) domains is observed. (**c**) *I*-*V* characteristic of junctions of Pt/LCMO/Nb:STO with a LCMO barrier of 2 nm and 4 nm and without barrier taken at 10 K. The schematic of the device and measurement configuration is depicted in the inset.

LCMO epitaxial thin films have been prepared by using RF magnetron sputtering on top of (001)Nb:STO substrates and capped by Pt. The films exhibit excellent microstructural qualities, a sharp interface with the substrate^28^ and a smooth surface with a terrace-step landscape (Fig. 1a). The films show bulk-like magnetic properties with only a small reduction of *T*_*C*_ in the thinnest samples (see Fig. S1). As reported in our previous work, films grown on SrTiO_3_ substrates are under tensile strain and exhibit strong PMA^21^. Magnetic properties of the barrier are not clearly determined via SQUID magnetometry because magnetic signal of such a thin film is very low. Thus, in our junctions, PMA has been proved by low-temperature (77 K) magnetic force microscopy (MFM) imaging (Fig. 1b). We visualize the zero-magnetization ferromagnetic domain configuration in the virgin state, after zero-field cooling. The greater part is composed by an almost equal distribution of up (blue color) and down (red color) domains, confirming that the magnetization is perpendicular to the surface. The thin white regions (indicating in-plane magnetization) correspond to the domain walls.

Transport measurements were performed in pillar structures by using a two-probe configuration setup as that sketched in Fig. 1c (inset). *I*-*V* curves performed on Pt/LCMO/Nb:STO tunneling junctions and a Pt/Nb:STO (without the insulating LCMO barrier) junction are depicted in Fig. 1c. The Pt/Nb:STO junction shows a rectifying behavior characteristic of a metal-semiconductor Schottky junction. As Nb:STO is an n-type semiconductor, forward bias corresponds to electrons being injected from the semiconductor to the metal electrode^29^. The insertion of the LCMO insulating barrier implies an increase of the barrier height, i.e. an enhancement of the breakdown threshold voltage. Increasing the LCMO barrier thickness (*t*) further increases the barrier width. From the shift of the breakdown voltage evidenced in Fig. 1c, we conclude that the limiting factor for conduction in the Pt/LCMO/Nb:STO system must not be attributed to Nb:STO but to the LCMO barrier itself.

**Figure 2:**
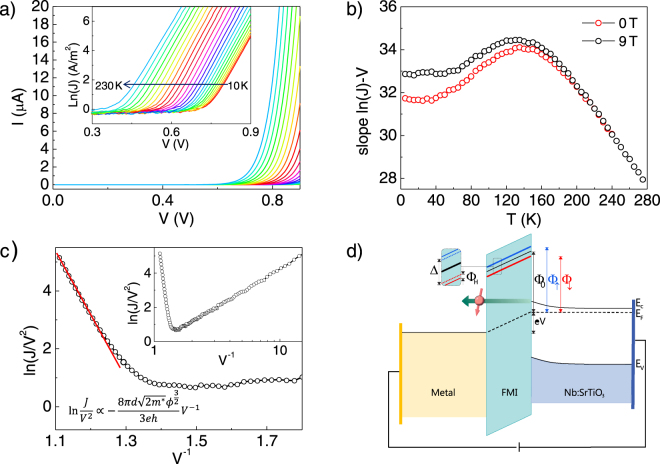
(**a**) *I*-*V* and corresponding ln(*J*)-*V* (inset) curves of the 2 nm-thick junction at different temperatures at zero magnetic field (temperatures from 10 K to 230 K every 10 K are represented). (**b**) ln(*J*)-*V* slope of the 2 nm-thick junction *I*-*V* curves as a function of temperature at zero field (red) and under 9 T OOP (black). (**c**) ln(*J*/*V*^2^)-*V*^−1^ plot from the curve at 10 K (from panel (a)) to determine the tunneling transport mechanism. The straight slope in the high voltage region indicates Fowler-Nordheim tunneling. (**d**) Schematics of the band diagram of our experimental configuration with forward applied bias. Φ_0_ is the tunneling barrier and splits into Φ_↑_ and Φ_↓_. In the zoom Δ is the exchange splitting and Φ_H_ is the additional splitting under an applied magnetic field.

The *I*-*V* characteristic curves of the 2 nm-thick junction measured at different temperatures are shown in Fig. 2a. The aforementioned rectifying behavior is observed over the whole range of temperatures (10–300 K). The behavior of the forward *I*-*V* characteristics is more clearly displayed as ln(*J*) vs. *V* (inset in Fig. 2a). It is evident that the curves are linear over several orders of magnitude. The temperature dependence of the slope of the ln(*J*) vs. *V* curves (with and without applied magnetic field) is then depicted in Fig. 2b. We observe that below 50 K the slope is almost constant, above this point it increases with temperature until reaching a maximum at about 150 K and then progressively decreases. According to the thermionic field emission theory the dominant transport mechanisms occurring in metal-insulator-metal junctions are thermionic emission of carriers over the potential barrier (by thermal activation at high temperature), and/or carrier tunneling through the barrier (field emission, at low temperature)^30,31^. In both cases, the current density depends exponentially on the barrier height and, for thermionic emission, also on the temperature. Then, the constant slope observed at low temperatures indicates that the dominant conduction process in this range is field emission, and thus probes tunneling through the barrier. In addition, the field emission process depends on the applied voltage. In fact, at high voltages, it is well described by Fowler-Nordheim tunneling (FNT). The tunneling current density in the FNT regime is given by the following equation^32^:

1 $$J=\frac{{e}^{3}{V}^{2}}{8\pi h{d}^{2}{{\rm{\Phi }}}_{0}}\exp (-\frac{d}{V}\frac{8\pi \sqrt{2{m}^{\ast }}{{\rm{\Phi }}}_{0}^{\frac{3}{2}}}{3eh})$$

where Φ_0_ is the barrier height, *h* is the Planck constant, *e* is the electron charge, m* is the effective mass of the electrons, *V* is the applied voltage and *d* is the thickness of the tunneling barrier. We can rewrite Eq. (1) as:

2 $${\rm{l}}{\rm{n}}\,\frac{J}{{V}^{2}}\propto \frac{8\pi d\sqrt{2{m}^{\ast }}{{\rm{\Phi }}}_{0}^{\frac{3}{2}}}{3eh}{V}^{-1}$$

The ln(*J*/*V* ^2^)-*V*^−1^ plot of the curve at *T* = 10 K is shown in Fig. 2d. The linear dependence in the high voltage bias regime is indicative of FNT and it is evident above 800 mV. The barrier height can be estimated from the slope obtaining a value of 1.2 eV.

Finally, in Fig. 2b, we also plot the slope of *I*-*V* measured under a magnetic field of 9 T. We observe that two set of measurements (without and with applied magnetic field) start to separate below *T*_*C*_ (≈190 K), indicating, as a first inspection, magnetoresistive effects.

### Magnetoresistance and spin-filtering effect

**Figure 3:**
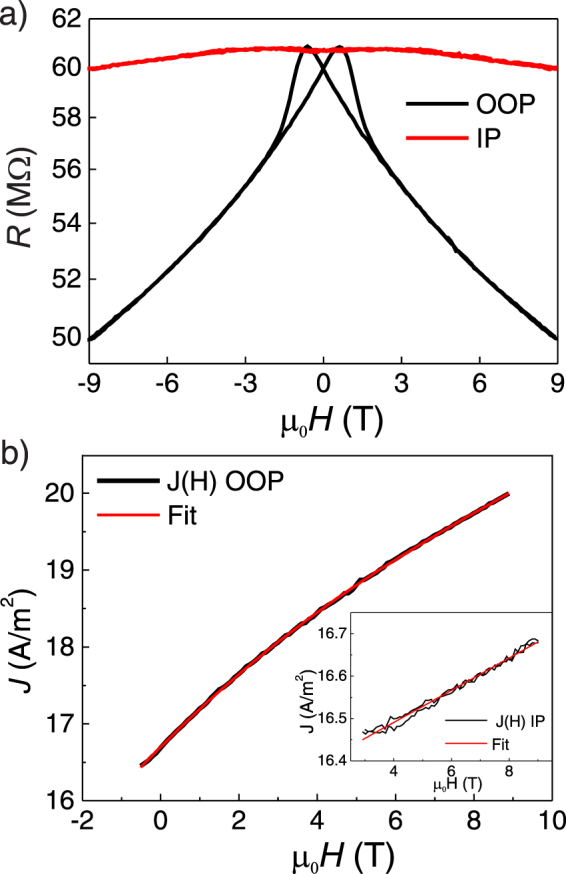
(**a**) Resistance versus field of a 200 *μ*m^2^ junction of Pt/2 nm-LCMO/Nb:STO with the field applied perpendicular and parallel to the sample at 10 K and *V* = 850 mV. (**b**) Fitting of *J*(*H*) to the two-current model extracted from measurements in (**a**) OOP field in main panel and IP field in the inset.

Now we explore is more detail the effects of the magnetic field on the transport properties of the junctions. The magnetic field dependence of the resistance of a 200 *μ*m^2^ junction with a 2 nm-thick LCMO barrier at 10 K is shown in Fig. 3a for a field applied both in-plane (IP) and out-of-plane (OOP).

As LCMO films exhibit strong PMA, the OOP case corresponds to the easy magnetization direction. With the field applied OOP the junction resistance exhibits a large negative magnetoresistance (MR) of about 20% at *μ*_0_*H* = 9T. The hysteretic behavior shown in the low field regime mimics the features of the magnetization curve: its maxima correspond to the coercive fields (see Fig. S1). In the IP case a much more modest value of the negative MR is found (2% for *μ*_0_*H* = 9T).

Finally, we have rule out the possibility that magnetoresistive effects are produced by spin-orbit interaction arising from Pt which is known to possess strong SOC. For that we have prepared junctions with Au instead of Pt as the top electrode. Au possesses a weaker SOC than Pt^33^ and it is a noble metal which will not give additional problems of oxidation, still, we have found very similar MR phenomenology (see Fig. S2). For completeness, we have also checked Pt/Nb:STO junctions (without LCMO) which do not present any MR effect.

To analyze the dependence of the MR on the applied magnetic field the two-current model was used^14,15^. According to this model spin-down and spin-up conduction bands of the LCMO lie at different energies due to the exchange splitting. When a magnetic field is applied those energies change in opposite directions and the tunneling barrier must be described as ${{\rm{\Phi }}}_{\uparrow (\downarrow )}={{\rm{\Phi }}}_{0}\pm {\rm{\Delta }}\mathrm{/2}\pm {{\rm{\Phi }}}_{H}$, where Φ_0_ is the average barrier height, Δ is the exchange splitting of the conduction band, and Φ_*H*_ accounts for the effect of magnetic field on this band.

**Figure 4:**
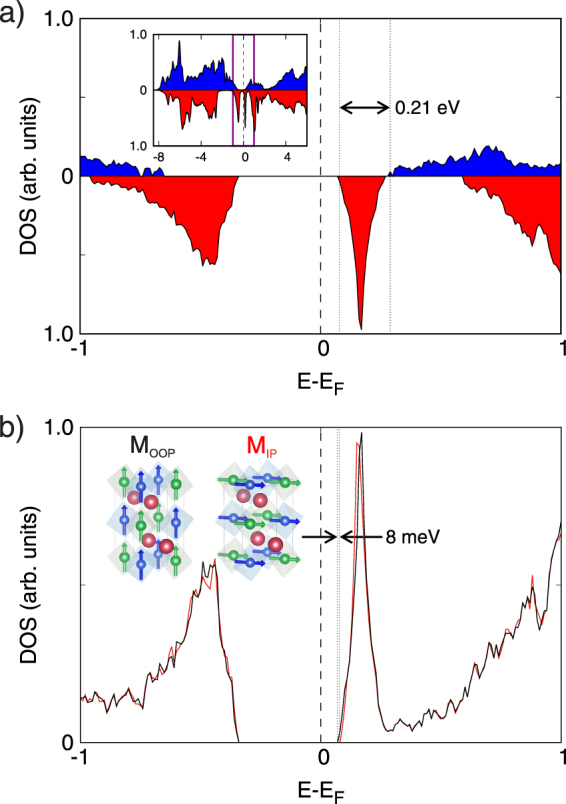
(**a**) Spin resolved DOS obtained by DFT calculations (red filled curve corresponds to spin down and blue one to spin up). The inset shows a view of a wider region in energy. (**b**) Total DOS obtained by DFT after forcing spins to be oriented in the (001)-direction (OOP, black line) and along (100)-direction (IP, red line). The conduction band minimum lies 8 meV lower when the magnetization points OOP, as indicated.

The band diagram of the system (with forward bias applied) is depicted in Fig. 2d. When a positive bias V is applied the Fermi level of the semiconducting electrode increases by eV^29^, accompanied by the corresponding reduction in the bending of the valence and conduction bands. According to two-current model the tunneling barrier Φ_0_ splits into Φ_↑_ and Φ_↓_ due to the exchange splitting Δ. At the same time spin-down and spin-up bands are modified by Φ_*H*_. The splitting of the conducting band position into majority and minority spin bands is confirmed by first principles calculations. The obtained spin-resolved density of states (DOS) are depicted in Fig. 4 and will be discussed in further detail later.

In the framework of the two-current model presented above, Eq. (1) describing the tunneling current density in FNT regime can be rewritten as:

3 $$J={J}_{\uparrow }+{J}_{\downarrow }=A[\frac{1}{{{\rm{\Phi }}}_{\uparrow }}\exp (-c{{\rm{\Phi }}}_{\uparrow }^{\frac{3}{2}})+\frac{1}{{{\rm{\Phi }}}_{\downarrow }}\exp (-c{{\rm{\Phi }}}_{\downarrow }^{\frac{3}{2}})]$$

where $A=\frac{{e}^{3}{V}^{2}}{8\pi h{d}^{2}}$ and $c=\frac{d}{V}\frac{8\pi d\sqrt{2{m}^{\ast }}}{3eh}$ and J_↑_(J_↓_) is the current density of spin up (down) electrons. In a rigid band approximation, the overall dependence of the Φ_↑_ and Φ_↓_ sub-bands on the applied magnetic field *H* (Φ_*H*_) should be described by a Zeeman energy term, namely Φ_*H*_ ≈ *μ*_0_*H*. This linear dependence and the fact that this term is small compared with Φ_0_ ± Δ/2 lead to a linear dependence of the *R*(*H*) curve^14^. Note in Fig. 3a that the dependence in the OOP case is clearly non-linear. Consequently, in the fitting of Eq. (3) to our experimental data (in OOP configuration) higher order terms in the field dependence of the barrier height have been taken into consideration.

The best fit results from testing ${{\rm{\Phi }}}_{H}=\alpha B+\beta {B}^{2}+\gamma {B}^{3}$, as shown in Fig. 3b, although an acceptable fit is obtained for ${{\rm{\Phi }}}_{H}=\alpha B+\beta {B}^{2}$. We also find an estimation of the Schotkky barrier of 1.27 eV for the 2-nm-thick barrier. The resulting value of the exchange splitting Δ is 0.2 eV, in good agreement with values reported in the literature^34^ and with our first principles calculations (0.21 eV). The coefficient for the linear term, *α* is around 1 meV/T. In contrast, the in-plane configuration actually exhibits the aforementioned linear dependence due to the Zeeman effect (see Fig. 3b (inset)). In this case, the fitted barrier height and exchange splitting are 1.27 eV and 0.2 eV, respectively, while we obtain *α* = 0.08 meV/T. The dependence on field of Φ_*H*_ in IP configuration is similar to the expected value for a pure Zeeman term (*μ*_*B*_ ~ 0.05 meV/T^15^). For the OOP configuration, it is twenty times bigger.

Explaining the strong difference in the field dependence of the junction resistance observed between the IP and OOP configurations is challenging. We tentatively attribute these differences to the magnetocrystalline anisotropy. Data regarding this remarkably different behavior are very scarce in the literature. Nevertheless, related phenomenology can be found in Fe_3_O_4_/spinel/La_2/3_Sr_1/3_MnO_3_ magnetic junctions with different compounds: CuCr_2_O_4_^12^ and CoCr_2_O_4_^35^). In these systems the magnetic anisotropy of the ferromagnetic insulating barrier seems to strongly affect the MR. CuCr_2_O_4_ is magnetically isotropic while CoCr_2_O_4_ presents strain-induced magnetocrystalline anisotropy^36^. In these systems, regions where the magnetization of the electrodes/spinel is constant (i.e. no switching of the electrodes/FM-barrier is involved) have very different dependence of the MR on the field. In the case of the CoCr_2_O_4_ barrier, with the magnetic field applied along the easy [001] direction Hu and Suzuki^35^ observe a non-linear variation of the MR of 10% within 4T. Comparatively, for the CuCr_2_O_4_ case with no magnetic anisotropy a variation of only 0.8% is found within 2T with a nearly linear dependence. Comparing with our experiment, we extract a variation of MR of 12% (OOP curve within 4T) and 0.5% (IP curve within 2T) from Fig. 2b.

To check the validity of our analysis we have explored the electronic structure of La_2_CoMnO_6_ by means of density functional theory (DFT) calculations within the local density approximation (LDA). When the SOC or the Hubbard terms are not included in the calculation, the system shows a metallic character meaning that La_2_CoMnO_6_ is a Coulomb-assisted SOC insulator. We have performed the calculations with a predetermined axis of magnetization (IP or OOP). The results obtained are depicted in Fig. 4.

The spin-resolved DOS for the magnetization perpendicular to the sample plane, OOP configuration, is shown in Fig. 4a. It renders an insulating ground state with a gap of ~0.4 eV. Moreover, the lowest available states in the conduction band are 0.21 eV higher for the spin up species than for the spin down species. The origin of the discrepancy between the obtained gap and the value estimated for the barrier height (1.27 eV) can be two-fold. On one hand, it is well know that DFT calculations within the LDA approximation suffer from a discontinuity in the derivative of the exact exchange correlation energy^37^, that drives to an underestimation of the band gap that can be as large as 80%^38,39^. On the other hand, the value of the electron correlation U (see Experimental Section for more details), must affect the gap.

In Fig. 4b we show the total DOS for the system with IP magnetization (red line) and OOP magnetization (black line). While the valence band maximum (VBM) is at the same energy in both cases, the conduction band minimum (CBM) for the OOP magnetization case lies 8 meV below its in-plane counterpart. The conduction band states with lower energy correspond to spin down Co states. Co states are strongly affected by spin-orbit interaction that is at the origin of this energy difference. Although, at a first glance this energy difference could seem very small, it is practically the same as the change in Φ_*H*_ between the IP and OOP configurations derived from the two-current model (9 meV at 9 T). In conclusion, theoretical calculations confirm that the direction of the magnetization must affect the tunnel resistance, thus predicting the existence of TAMR, and reinforcing the role of the magnetic anisotropy in this system.

On the other hand, the analysis of the tunneling current density in the FNT regime by using the two-current model, as given by Eq. (3), allows to estimate both *J*_↑_ and *J*_↓_ and to obtain the spin filtering efficiency of the LCMO barrier, *P*, which is given by:

4 $$P=\frac{{J}_{\uparrow }-{J}_{\downarrow }}{{J}_{\uparrow }+{J}_{\downarrow }}$$

Fitted parameters render nearly fully spin-polarized current with absolute polarization values above 99.7% for the 2 nm-thick sample.

### Angular dependence of the TAMR

**Figure 5:**
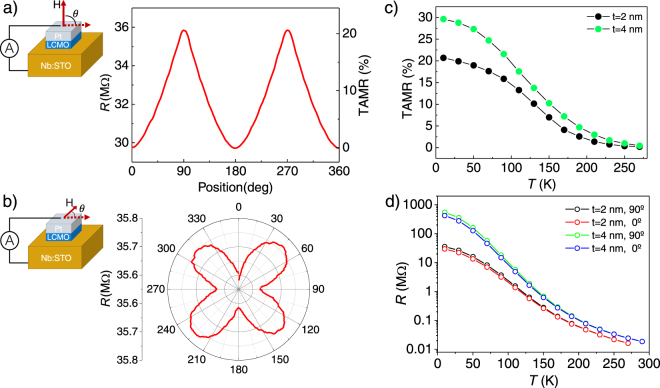
(**a**) Resistance and TAMR vs. *θ* (position of the sample) with the field rotating from OOP, *θ* = 0,180° to IP *θ* = 90,270°. (**b**) Resistance vs. rotation of the sample with the field applied IP. Both measurements were taken at 9 T, 10 K and *V* of 900 mV. Measurement configurations on the left. (**c**) TAMR vs. temperature (calculated from resistance vs. rotation curves) and (**d**) resistance vs. temperature of 2 nm and 4 nm-thick barrier samples with magnetic field applied OOP (red, blue) and IP (black, green).

The angular dependence of the tunneling junction resistance with respect to the direction of the applied magnetic field 2 nm-thick sample is depicted in Fig. 5a. The right axis of the plot corresponds to the TAMR ratio, which is defined as TAMR(*θ*) = [R(*θ*) − R(90°)]/R(90°), where *θ* is the position of the sample with respect to the field, and reveals a maximum variation of 20% in the tunneling resistance. The magnetic field is continuously rotated from OOP to IP. A magnetic field of 9 T, large enough to ensure full saturation of the magnetization in the field direction, has been used. The expected uniaxial anisotropy is found with the junction resistance reaching a minimum when the magnetic field is applied along the easy axis (*θ* = 0,180°).

The significant correlation between resistance and the orientation of the sample with respect to the magnetic field, confers the device possibilities to be used as an angular position or rotation sensor. The most commonly used material for sensor using anisotropic magnetoresistance effect is permalloy, which typically has anisotropic magnetoresistance (also known as magnetoresitive coefficient) of 1.5–3%^40^. Therefore, in our device, the magnetoresistive coefficient reaches 20%. Additionally from Fig. 5a we determine a sensitivity of 3 nA/rad. For magnetic field detection, from Fig. 3a, we obtain sensitivity around 3 nA/T when the magnetic field is parallel to the easy axis of the sensor, thus perpendicular to it. These sensitivity values could be triggered by changing the lateral size of the device.

We have also analyzed the angular dependence of the tunneling junction resistance when the magnetic field is rotated within the plane of the film, as depicted in Fig. 5b. In this configuration the tunneling current is perpendicular to the magnetic field that is rotated. We obtain a fourfold dependence of the TAMR. The resistance is minimum when the magnetic field is applied along the (100) and (010) crystallographic directions of Nb:STO, corresponding to the (110) and (1-10) directions of LCMO. This result indicates the existence of two in-plane easy magnetization axes along these directions. Nevertheless the TAMR found in the IP configuration is around 1%, much smaller than in the OOP to IP case (20%), confirming the relevance of the system PMA in TAMR.

The temperature dependence of the TAMR (OOP-to-IP case) has further been analyzed. TAMR values have been calculated from resistance vs. position curves at different temperatures (see Fig. 5c). The TAMR response smoothly decreases with increasing temperature, and vanishes close to *T*_*c*_, thus demonstrating that it is linked to the magnetic state of the FM-I barrier.

We have also investigated the TAMR in the 4 nm-thick barrier heterostructure (see Fig. S3). The very same anisotropic behavior is found irrespective of *t* as shown in Fig. 5c, rendering values of about 20% for *t* = 2 nm and 30% for *t* = 4 nm at *T* = 10 K. Besides, the difference in resistance between the *t* = 2 nm and *t* = 4 nm barriers is of one order of magnitude (see Fig. 5d), proving the exponential dependence of the tunneling current on *t*.

Additionally, we have inspected the TAMR voltage dependence. It shows a maximum response at *V* of 900 mV and then progressively decreases as the applied voltage increases. This reduction of the TAMR as the voltage increases is expected for the FNT regime and the double channel expressions. A similar behavior is generally found in MTJs and is usually attributed to spin excitations localized at the interfaces between the magnetic electrodes and the tunnel barrier^41^).

### Memory device

**Figure 6:**
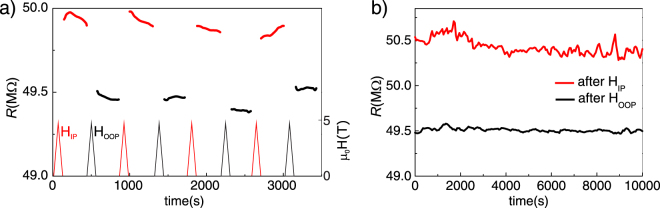
(**a**) Resistance measured during 300 s after applying/releasing a field of 5 T along the OOP direction (black) and IP direction (red). Schematic of magnetic field pulses is depicted at the bottom of the figure. (**b**) Long time measurement (10^5^ s) of the two resistance states after a 9 T pulse OOP and IP.

Revisiting Fig. 3a, we observe a clear difference between the zero-field resistance of the IP and OOP configurations. We examine the possibility of designing a memory device where two distinct remanent states could be obtained by changing the orientation of the applied magnetic field. Hence, we have performed successive cycles of applying/releasing field along the IP and OOP directions, followed by a measurement of the resistance at zero applied field. The results are depicted in Fig. 6a. Two resistance states are clearly differentiated and reproducible over several repetitions. The stability of those states has been tested by measuring the relaxation of the resistance for long periods of time, as it is shown in Fig. 6b. We found that the variations in the resistance after relaxation times of about 10^5^ s are nearly negligible in both cases, thus demonstrating high retention power. Therefore, Pt/LCMO/Nb:STO structures present two well defined stable memory states separated by up to 1–2% in resistance. The magnitude of this effect is small but comparable to other bistable resistance memory systems^42^. Nonetheless this system is unique and much simpler, in the sense that it is based on only one magnetic layer and TAMR capabilities.

## Conclusions

In summary, we report a spintronic device based on a single magnetic insulating layer. The studied heterostructures consist of a thin LCMO layer grown on top of Nb:STO substrate and capped with a thin layer of metal (Pt or Au). LCMO, being a ferromagnetic insulating material, possesses strong PMA. We have found that the tunneling resistance of the device strongly depends on the relative orientation between the magnetic field and the easy magnetization axis. Our results evidence a strong non-linear dependence of the resistance on the applied field when it is applied along the easy axis (OOP). As a result, the tunneling MR is highly anisotropic leading to TAMR values as high as 20–30% at low temperature when the magnetization is rotated from OOP to IP. These results are corroborated by DFT-based calculations. We demonstrate that the DOS of its parent compound La_2_CoMnO_6_ has a fully polarized spin-down character above the Fermi level. On the other hand, we estimate a difference in the tunnel barrier height of 8 meV when magnetization changes from OOP to IP, and an exchange splitting of 0.2 eV, in good agreement with values fitted experimentally. In addition, exchange splitting and barrier height values found by this fitting lead to an estimation of the spin filtering efficiency of almost 100%.

Lastly, we probe the existence of a non-volatile bistable resistive state that can be switched by applying magnetic field pulses in perpendicular or parallel directions. Notwithstanding that our device works below room temperature and using high magnetic fields, it represents, to the best of our knowledge, the first report of a single layer ferromagnetic memory with electrical read-out.

We have presented an attractive approach for a new class of spintronic devices: a magnetic sensor and memory based on FM-I with strong perpendicular magnetic anisotropy. These findings open a new platform for exploring other highly anisotropic FM-I materials with higher transition temperatures.

## Experimental Section and Calculation Details

### Sample Fabrication and Characterization

Pt(Au)/LCMO/Nb(0.7%):STO (001) heterostructures have been fabricated by RF magnetron sputtering with different LCMO insulating barrier thickness (2 and 4 nm and without barrier). LCMO films have been grown at 900°C with an oxygen partial pressure of 0.4Torr, *in situ* thermal annealing after deposition in oxygen atmosphere and slow cooling rate of 10°C/min. A layer of 3–4 nm of Pt(Au) has been deposited *in situ* after cooling. Photolithography has been used to pattern 100 to 400 *μ*m^2^ tunnel junctions, ion milling was used to etch the material and penetrate until the substrate to define pillar structures. Electrical insulation between different tunnel junctions has been accomplished by depositing a photoresist, conducting paths to the junction were opened with a microwriter and Pt macrocontacts have been deposited with a shadow mask.

The smoothness of the surface was checked by atomic force microscopy (AFM), and (MFP-3D AFM, Asylum Research). Magnetic properties of LCMO thin films have been analyzed by using SQUID magnetometry (see SI). For *t* = 2 and 4 nm a reduction of *T*_*C*_, down to about 190 K, is detected. To study the magnetic domain distribution Magnetic Force Microscopy (MFM) image at low temperature (77 K) was performed using an AttoAFM I (Attocube Systems) and commercial ASYMFMHC (Oxford Instruments) coated with CoPt/FePt (30 nm).

*I*-*V* curves were measured by using a two terminal (Nb:STO negative, Pt (Au): positive) setup in a commercial physical property measurement system (PPMS, Quantum Design) in pillar structures. To avoid Joule heating effects the measuring currents were limited to 1000 *μ*A. The current was applied perpendicular to the film plane while the magnetic field was rotated from out-of-plane, i.e. parallel to the [001] direction in which the current flows, to in-plane parallel to [100] direction. In-plane anisotropy was also explored by rotating the magnetic field in the sample plane while keeping it perpendicular to the tunneling current.

### Theory

Density functional theory (DFT) calculations have been carried out within the local density approximation (LDA) as implemented in the SIESTA-GREEN package^43,44^, employing norm-conserving Troullier-Martins pseudopotentials^45^. The electron-electron interaction is introduced through the LDA+ U approach^46,47^, which describes electron correlations through a single effective parameter U_*eff*_ = U − J. We set U_*eff*_ to 3.0 eV for Co and Mn *d* shells, as usually employed in the literature for similar compounds^48–50^. SOC was taken into account in the calculations following the self-consistent fully-relativistic pseudo-potential formalism implemented within the SIESTA framework^51^. Self-consistent DOS calculations were performed in a 12 × 12 × 9 *k*-point grid using a fine mesh cutoff of 1000 Ry for three-center integrals. We employed a double-*ζ* polarized basis set with confinement energies of 100 meV, and the electronic temperature in the Fermi-Dirac distribution was set to *k*_*B*_*T* = 10 meV in the calculations.

## Electronic supplementary material

Supplementary Information
