# Supplementary material for: Anisotropic sensor and memory device with a ferromagnetic tunnel barrier as the only magnetic element
Source: Sci Rep. 2018 Jan 16;8:861. doi: 10.1038/s41598-017-19129-5 (PMC5770439; doi:10.1038/s41598-017-19129-5)
Supplement: Supplementary file 1 — Supplementary Information [file 41598_2017_19129_MOESM1_ESM.doc]

*Supplementary Information*

**Anisotropic sensor and memory device with a ferromagnetic tunnel barrier as the only magnetic element**

L. López-Mir, C. Frontera, H. Aramberri, K. Bouzehouane

J. Cisneros-Fernández, B. Bozzo, Ll. Balcells, and B. Martínez.

The magnetization of very thin films of LCMO grown on STO (down to 4 nm) has been published previously by the authors.^[1]^ In Figure S1 we present the magnetic properties of Pt/LCMO(9nm)/Nb:STO, measured by applying the field in the out of plane.

| 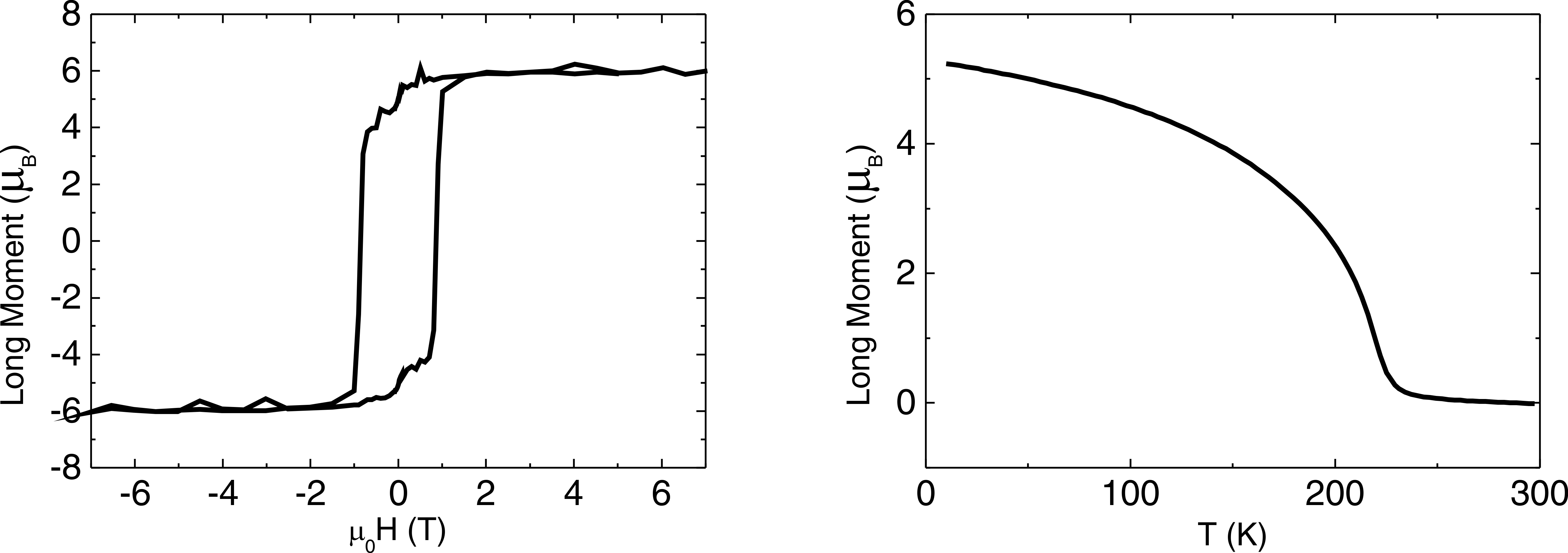 |
| --- |
| **Figure S1.** Magnetic properties of a 9 nm thick Pt/LCMO/Nb:STO. The film exhibits bulk –like saturation magnetization, i.e. 6 μ_B_/f.u. (Left: M(H) curve at T= 10 K), but with a slightly reduced transition temperature of 220 K (T_C_ ≅ 230 K ) (Right: Temperature dependence of the magnetization after a zero field cooling – field cooling process. H= 1000 Oe). In both panels, magnetic field is applied out of plane*.* |

We have studied the magnetotransport properties of our system by using gold as electrode material. Results are plotted in Figure S2. In comparison with Pt, Au has much smaller spin orbit coupling. Besides small changes in the overall values, the general tendency in Au/LCMO/Nb:STO is nearly identical to that in Pt/LCMO/Nb:STO.

| 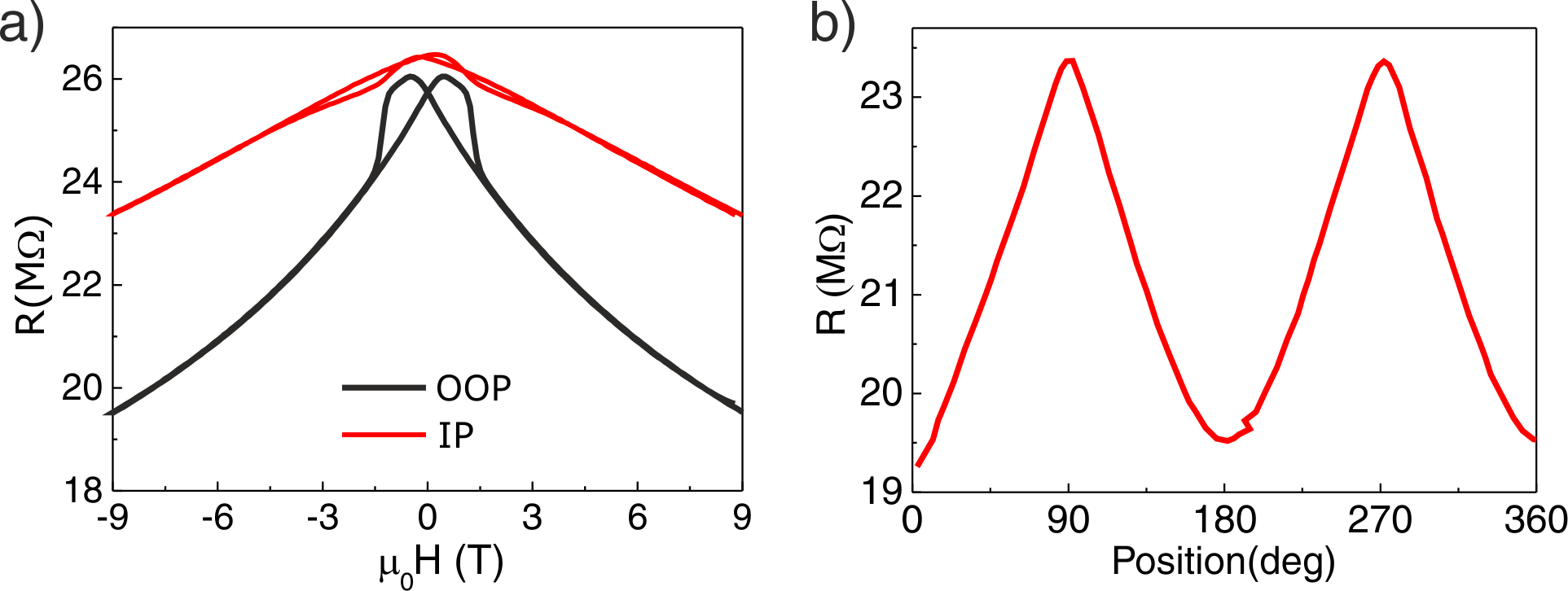 |
| --- |
|  |
| **Figure S2**. a) Magnetic field dependence of the resistance of a 200 μm^2^ junction of Au/2nm-La_2_CoMnO_6_/Nb:SrTiO_3_ with the field applied perpendicular (black) and parallel(red) to the sample plane. b) Dependence of the resistance on the relative orientation of the sample with respect to the magnetic field of 9T*.* In both panels, data were taken at *T*= 10 K and applying *V*= 800 mV. |

Finally, we present the results corresponding to the 4nm barrier: Pt/LCMO(4nm)/Nb:STO.

| 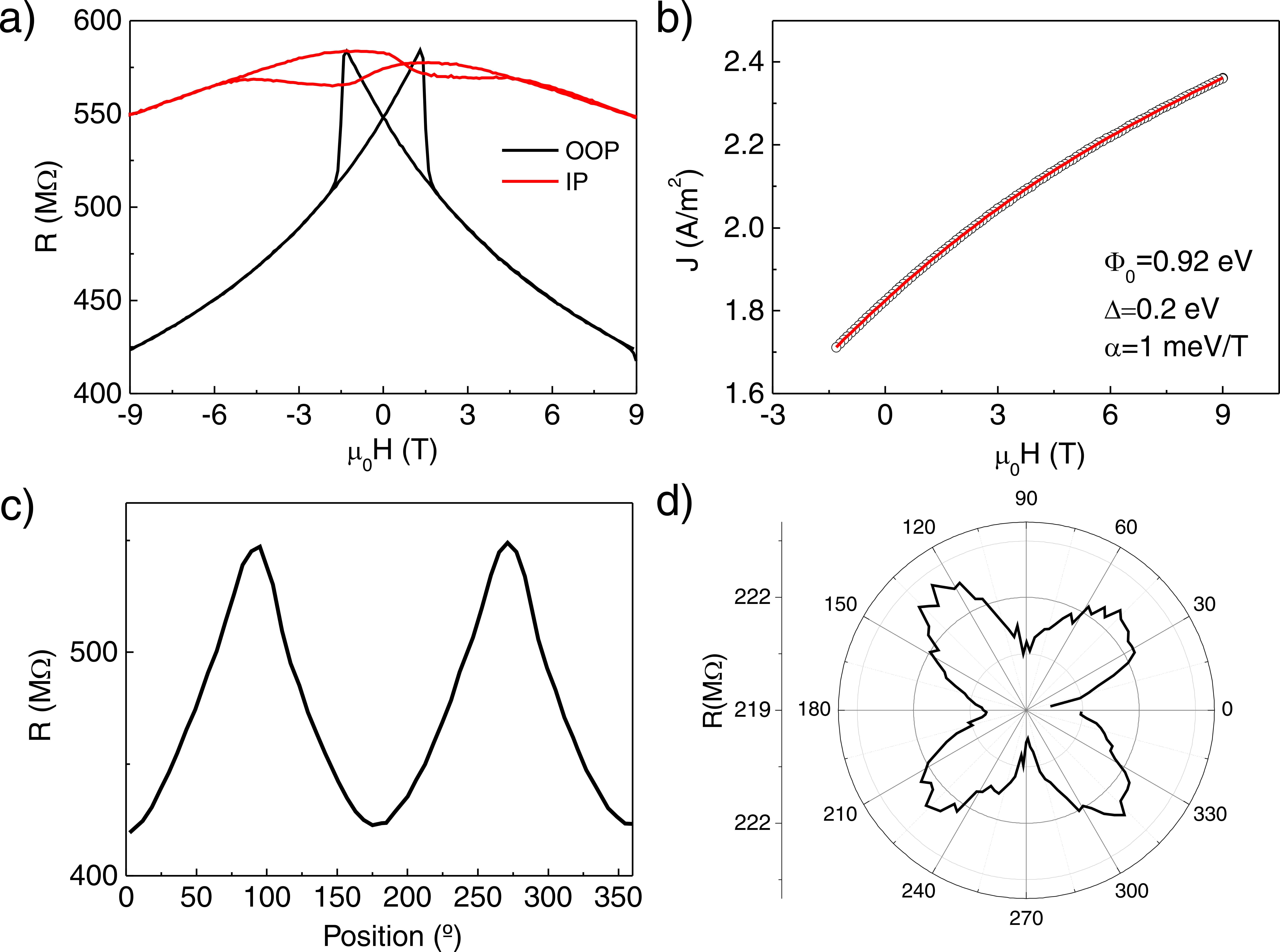 |
| --- |
| **Figure S3**. a) Resistance versus field of a 120 μm^2^ junction of Pt/4nm-La_2_CoMnO_6_/Nb:SrTiO_3_ with the field applied perpendicular the sample at 10K an applied bias voltage of 900 mV. (b) Fitting of J(H) to two-current model extracted from measurements in (a) for the OOP case. (c)Resistance versus position of the sample with respect to the magnetic field of 9 T, when this is rotated from OP(0º,180º) to IP (90º,270º). (d) Polar plot of the resistance when the magnetic field (9 T) is rotated IP. |

References:

[1] R. Galceran, L. López-Mir, B. Bozzo, J. Cisneros-Fernández, J. Santiso, L. Balcells, C. Frontera, B. Martínez*, Phys. Rev. B - Condens. Matter Mater. Phy*s**. 20**16*,* 93.
